# MicroRNA deregulation in nonalcoholic steatohepatitis-associated liver carcinogenesis

**DOI:** 10.18632/oncotarget.19774

**Published:** 2017-08-01

**Authors:** Aline de Conti, Juliana Festa Ortega, Volodymyr Tryndyak, Kostiantyn Dreval, Fernando Salvador Moreno, Ivan Rusyn, Frederick A. Beland, Igor P. Pogribny

**Affiliations:** ^1^ Division of Biochemical Toxicology, National Center for Toxicological Research, Jefferson, Arkansas, USA; ^2^ Department of Food and Experimental Nutrition, Faculty of Pharmaceutical Sciences, University of São Paulo, São Paulo, Brazil; ^3^ Department of Veterinary Integrative Biosciences, Texas A&M University, College Station, Texas, USA

**Keywords:** hepatocarcinogenesis, non-alcoholic steatohepatitis, epigenetics, microRNAs, miR-93-5p

## Abstract

Hepatocellular carcinoma (HCC) is the fastest-rising cause of cancer-related death in the United States. Recent epidemiological studies have identified nonalcoholic steatohepatitis (NASH), a progressive form of nonalcoholic fatty liver disease (NAFLD), as a major risk factor for HCC. Elucidating the underlying mechanisms associated with the development of NASH-derived HCC is critical for identifying early biomarkers for the progression of the disease and for treatment and prevention. In the present study, using liver samples from C57BL/6J mice submitted to the Stelic Animal Model (STAM) of NASH-associated liver carcinogenesis, we investigated the role of microRNA (miRNA) alterations in the pathogenesis of NASH-derived HCC. We found substantial alterations in the expression of miRNAs, with the greatest number occurring in full-fledged HCC. Mechanistically, altered miRNA expression was associated with activation of major hepatocarcinogenesis-related pathways, including the TGF-β, Wnt/β-catenin, ERK1/2, mTOR, and EGF signaling. In addition, the over-expression of the miR-221-3p and miR-222-3p and oncogenic miR-106b∼25 cluster was accompanied by the reduced protein levels of their targets, including E2F transcription factor 1 (E2F1), phosphatase and tensin homolog (PTEN), and cyclin-dependent kinase inhibitor 1 (CDKN1A). Importantly, miR-93-5p, miR-221-3p, and miR-222-3p were also significantly over-expressed in human HCC. These findings suggest that aberrant expression of miRNAs may have mechanistic significance in NASH-associated liver carcinogenesis and may serve as an indicator for the development of NASH-derived HCC.

## INTRODUCTION

Nonalcoholic fatty liver disease (NAFLD), the hepatic manifestation of the metabolic syndrome, and nonalcoholic steatohepatitis (NASH), an advanced form of NAFLD, are becoming dominant chronic liver diseases and major health problems in the United States and Western countries [1, 2]. Furthermore, the results of extensive epidemiological studies have established convincingly an association between NASH and the development of hepatocellular carcinoma (HCC) in humans [3, 4].

The sequential accumulation of pathomorphological alterations during the natural progression of NASH to HCC is well characterized, whereas the underlying molecular pathophysiological mechanisms of NASH-related liver carcinogenesis have not been defined. Uncovering the pathogenesis of this process is critical for effective disease diagnosis, treatment, and prevention.

Comprehensive research efforts in recent years have documented an aberrant level of microRNAs (miRNAs) in liver tissue and systemic circulation of patients with NASH and HCC [5–8]. Based on this evidence, it has been proposed that miRNAs may have diagnostic and prognostic significance and, more importantly, be attractive therapeutic targets and therapeutic molecules for HCC treatment. Furthermore, it has been suggested that aberrant expression of miRNAs in hepatic tissue may be related to the disease pathogenesis; however, there is insufficient knowledge to clarify the role and mechanism of miRNA alterations with respect to the carcinogenic process, including NASH-related hepatocarcinogenesis. The detection of an abnormal expression of any given hepatic miRNA in NASH or HCC is not sufficient to address decisively the role of the miRNA alteration in the pathogenesis of the disease, since it may be a secondary consequence of the pathomorphological state. To provide evidence of an association between miRNA alterations and the development of NASH-related HCC, it is necessary to demonstrate, at least, (*i*) that the altered expression of the miRNA occurs at the preneoplastic stages of liver carcinogenesis; (*ii*) that miRNA alterations occurring in preneoplastic lesions persist, along with the accumulation of additional miRNA alterations, in full-fledged HCC; and (*iii*) there is a mechanistic link between miRNA alterations and HCC development. To address these questions using human samples is frequently unpractical and typically very complex, whereas relevant animal models that resemble the development of HCC in NASH patients largely compensate the limitations of human-only studies.

Based on these considerations, the goal of the present study was to investigate the role of miRNA deregulation in the pathogenesis of NASH-associated liver carcinogenesis by using the Stelic Animal Model (STAM) of liver carcinogenesis, a mouse model of NASH-related HCC that resembles the development of the disease in humans [9, 10]. In the present study, we examined the miRNA expression profiles in the livers of STAM mice at steatotic (6 weeks), NASH-fibrotic (12 weeks), and full-fledged HCC (20 weeks) stages of liver carcinogenesis. The results of this study demonstrate substantial alterations in miRNA expression during NASH-associated liver carcinogenesis, with the greatest number of differentially expressed miRNAs being found in full-fledged HCC. Furthermore, we identified 19 differentially expressed miRNAs that exhibited ≥ 2-fold expression change in HCC. Among these miRNAs, the over-expression of ten miRNAs (miR-15b-5p, miR-17-5p, miR-25-3p, miR-93-5p, miR-106b-5p, miR-181b-5p, miR-186-5p, miR-221-3p, miR-222-3p, and miR-223-3p) was associated with the activation of major hepatocarcinogenesis-related pathways, including the TGF-β, Wnt/β-catenin, ERK1/2, mTOR, and EGF signaling. Furthermore, these miRNAs exhibited increased expression during carcinogenic progression from the NASH stage to full-fledged HCC. We also demonstrated that over-expression of miR-25-3p, miR-93-5p, miR-106b-5p, miR-221-3p, and miR-222-3p was accompanied by the reduced protein levels of their targets, including E2F1, PTEN, and CDKN1A. Importantly, miR-93-5p, miR-221-3p, and miR-222-3 were significantly over-expressed in human HCC. These findings suggest that aberrant expression of these miRNAs may serve as an indicator of the progression of the hepatocarcinogenic process.

## RESULTS

### miRNA expression alterations in the livers of STAM mice

**Figure 1:**
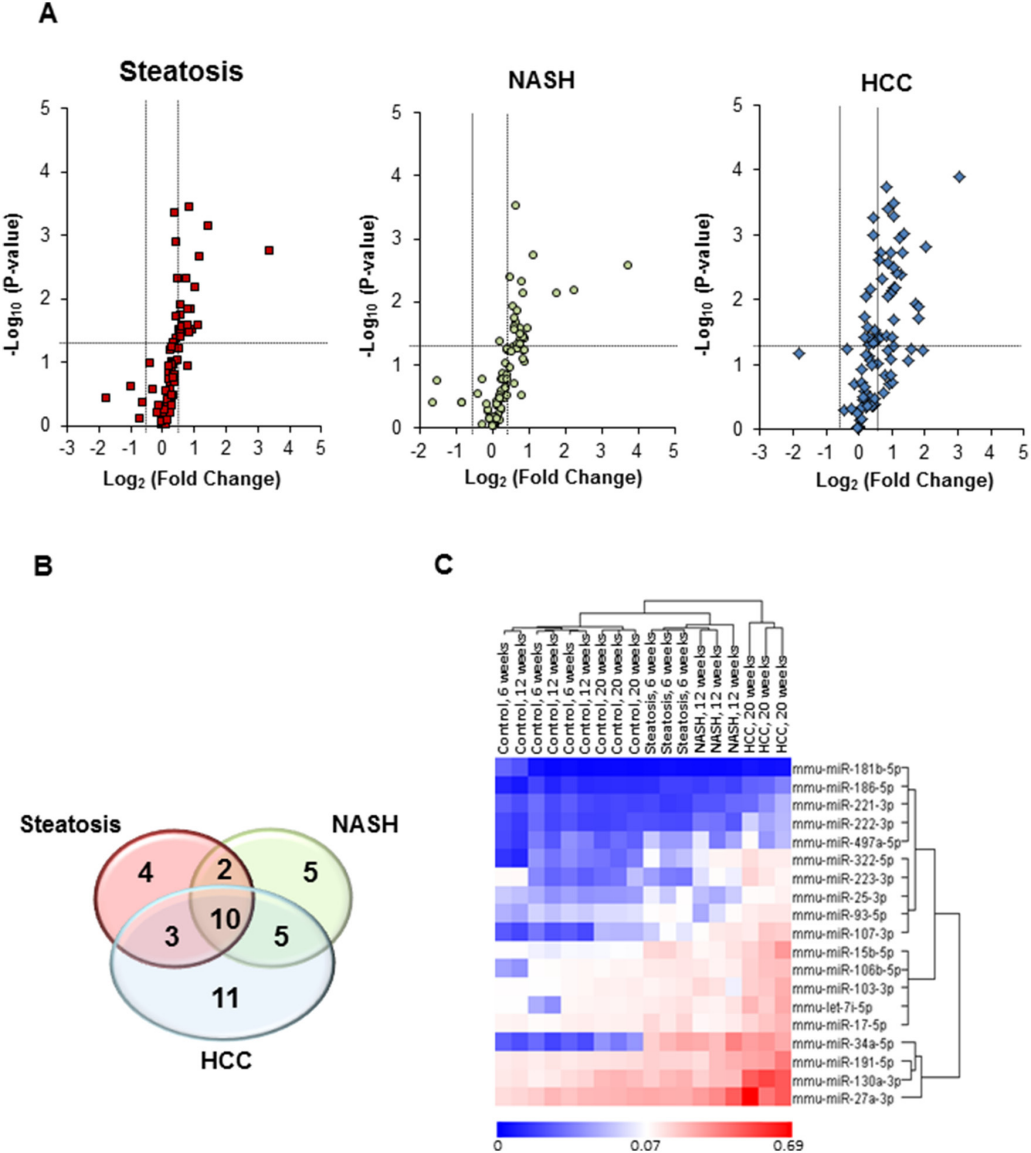
miRNA expression profiles in the livers of STAM mice subjected to NASH-associated hepatocarcinogenesis **(A)** miRNA expression profiles in the livers of STAM mice at steatosis (6 weeks), NASH-fibrosis (12 weeks), and full-fledged HCC (20 weeks) stages of NASH-related liver carcinogenesis relative to control mice (n = 4 in control and experimental groups). The base 2-logarithm of the fold change is plotted on the X-axis, and the negative of the base 10-logarithm of the P-value is plotted on the Y-axis. Vertical black-dotted lines indicate the cut-off values for a 1.5-fold change. Horizontal black-dotted line indicates a P-value of 0.1. **(B)** Venn diagram illustrating number of differentially expressed miRNAs at 6, 12, and 20 weeks in the livers of STAM mice subjected to NASH-derived hepatocarcinogenesis. **(C)** Heat maps and unsupervised hierarchical cluster analysis of the 2^*−ΔΔCt*^ values illustrating differences in the expression of ≥ 2-fold hepatic miRNA profiles between control mice and STAM mice at steatosis (6 weeks), NASH-fibrosis (12 weeks), and full-fledged HCC (20 weeks) stages of NASH-related liver carcinogenesis. Color bar identifies high-expressed (red) and low-expressed (blue) miRNAs.

**Figure 2:**
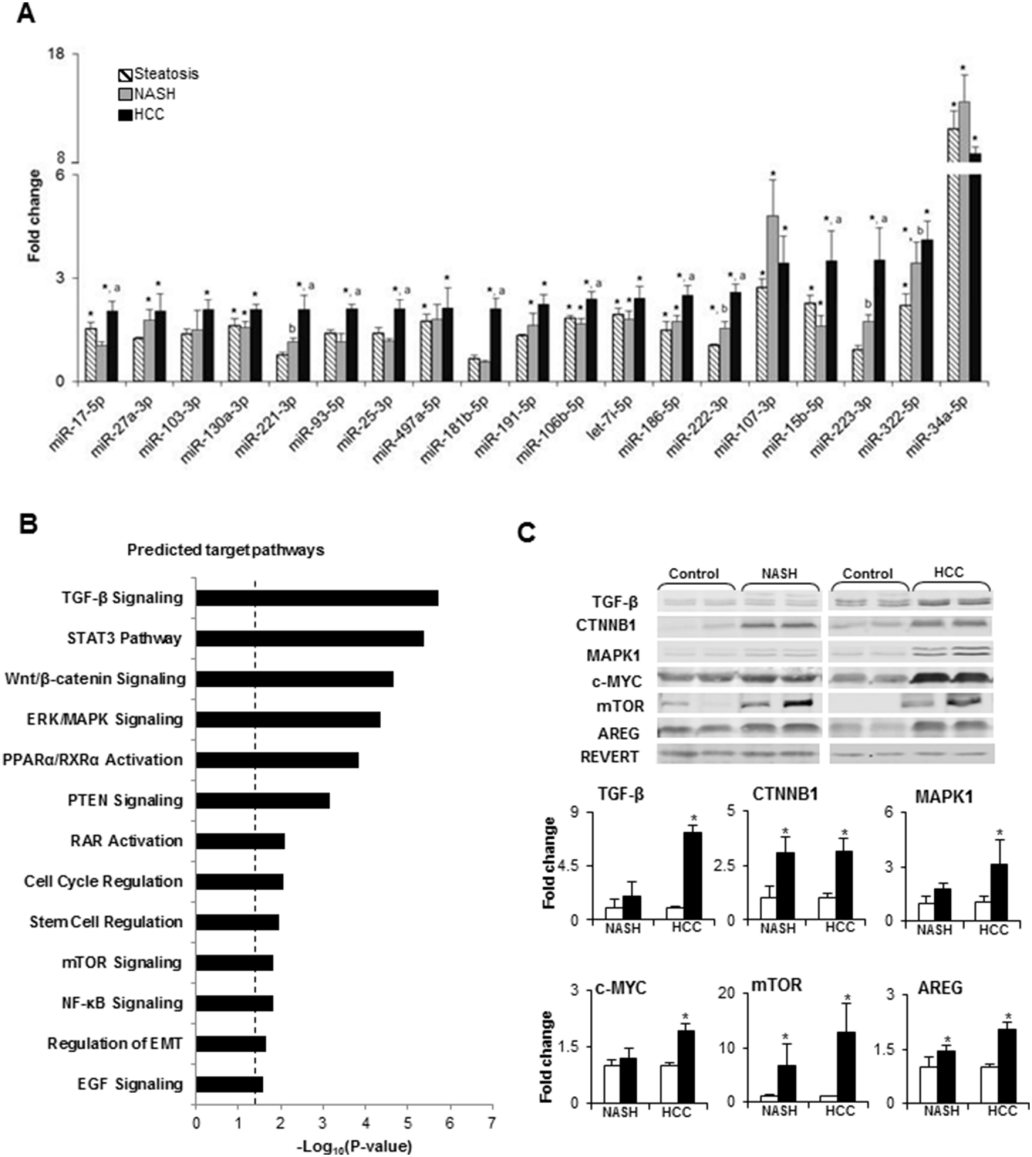
Summary of deregulated molecular pathways associated with differentially expressed miRNAs in NASH-derived HCC **(A)** Dynamics in the expression changes of HCC-specific miRNAs during NASH-associated liver carcinogenesis. * - denotes a significant (P < 0.05) difference from the age-matched control group; ^a^ - denotes a significant (P < 0.05) difference from the NASH-fibrosis stage; ^b^ - denotes a significant (P < 0.05) difference from the steatosis stage. **(B)** Molecular pathways affected by differentially expressed miRNAs in HCC. P-Values were calculated using IPA to predict biological functions. **(C)** The protein level of TGF-β, CTNNB1, MAPK1, c-MYC, mTOR, and AREG, key members of deregulated miRNA-associated molecular pathways, identified by IPA, in the livers of control mice and STAM mice at NASH-fibrosis and full-fledged HCC stages of hepatocarcinogenesis. The results are presented as an average fold change in the level of each protein in the livers of STAM mice relative to that in the age-matched control mice, which were assigned a value 1. *White bars* – control mice; *black bars* - STAM mice. Values are mean ± SD, n = 5. * - denotes a significant (P < 0.05) difference from the age-matched control group. Representative Western blot images of 2 different samples from the each group are shown.

While the significance of the stepwise accumulation of genetic and epigenetic alterations in the development of HCC has been extensively studied [11, 12], the role of miRNA alterations in the process of liver carcinogenesis has remained largely unexplored, despite of multiple studies reporting aberrant expression of miRNAs in HCC [5–8, 13]. Hence, miRNA expression profiles were examined in control, steatotic (6 weeks), NASH-fibrotic (12 weeks), and full-fledged HCC (20 weeks) liver samples. The results of the miRNAs expression analyses showed progressive alterations of the hepatic miRNome during the development of NASH-derived HCC, as evidenced by an increasing number of differentially expressed (fold change ≥ 1.5 and Benjamini-Hochberg adjusted P< 0.1) miRNAs, 19, 22, and 29 miRNAs, respectively, at steatotic, NASH-fibrotic, and HCC stages of carcinogenesis (Figures 1A and 1B, Supplementary Table 1). Among these miRNAs, 10 were expressed in common at all stages (Figures 1A and 1B, Supplementary Table 1). The greatest number of differentially-expressed miRNAs was found in full-fledged HCC (Supplementary Table 1, Figures 1A, 1B and 1C), with 19 of the miRNAs being differentially expressed ≥ 2-fold in full-fledged HCC (Supplementary Table 1 and Figures 1C and 2A). Among these 19 differentially expressed miRNAs in HCC (20 weeks), 10 of the miRNAs were significantly different from that in NASH-fibrotic livers (12 weeks), among which miR-221-3p, miR-222-3p, and miR-223-3p showed a progressive stage-dependent increase (Figure 2A). This suggests that these miRNAs may have significance in the transition of preneoplastic cells into cancerous cells. Indeed, the IPA demonstrated the involvement of these 10 miRNAs in major liver carcinogenesis-related pathways, [14] including TGF-β, STAT3, Wnt/β-catenin, ERK/MAPK, PPARα/RXRα, PTEN, RAR, cell cycle regulation, stem cell regulation, mTOR, and NF-κB signaling, EMT, and EGF (Figure 2B).

In order to confirm the results of the IPA software, the protein level of key members of pathways that were identified by IPA was analyzed by Western blotting. Figure 2C shows a significant increase in the levels of transforming growth factor β (TGF-β), β-catenin (CTNNB1), extracellular signal–regulated kinase 1 (MAPK1), c-MYC, mechanistic target of rapamycin (mTOR), and amphiregulin (AREG) in HCC tissue samples, confirming independently the activation of these cancer-related pathways in HCC. Moreover, the level of CTNNB1, mTOR, and AREG was also increased by 3-, 6-, and 1.4-times in NASH/fibrotic liver tissue, suggesting activation of the Wnt/β-catenin, mTOR, and EGF signaling pathways at the preneoplastic stage of hepatocarcinogenesis.

### Expression of miRNAs in human HCC

**Figure 3:**
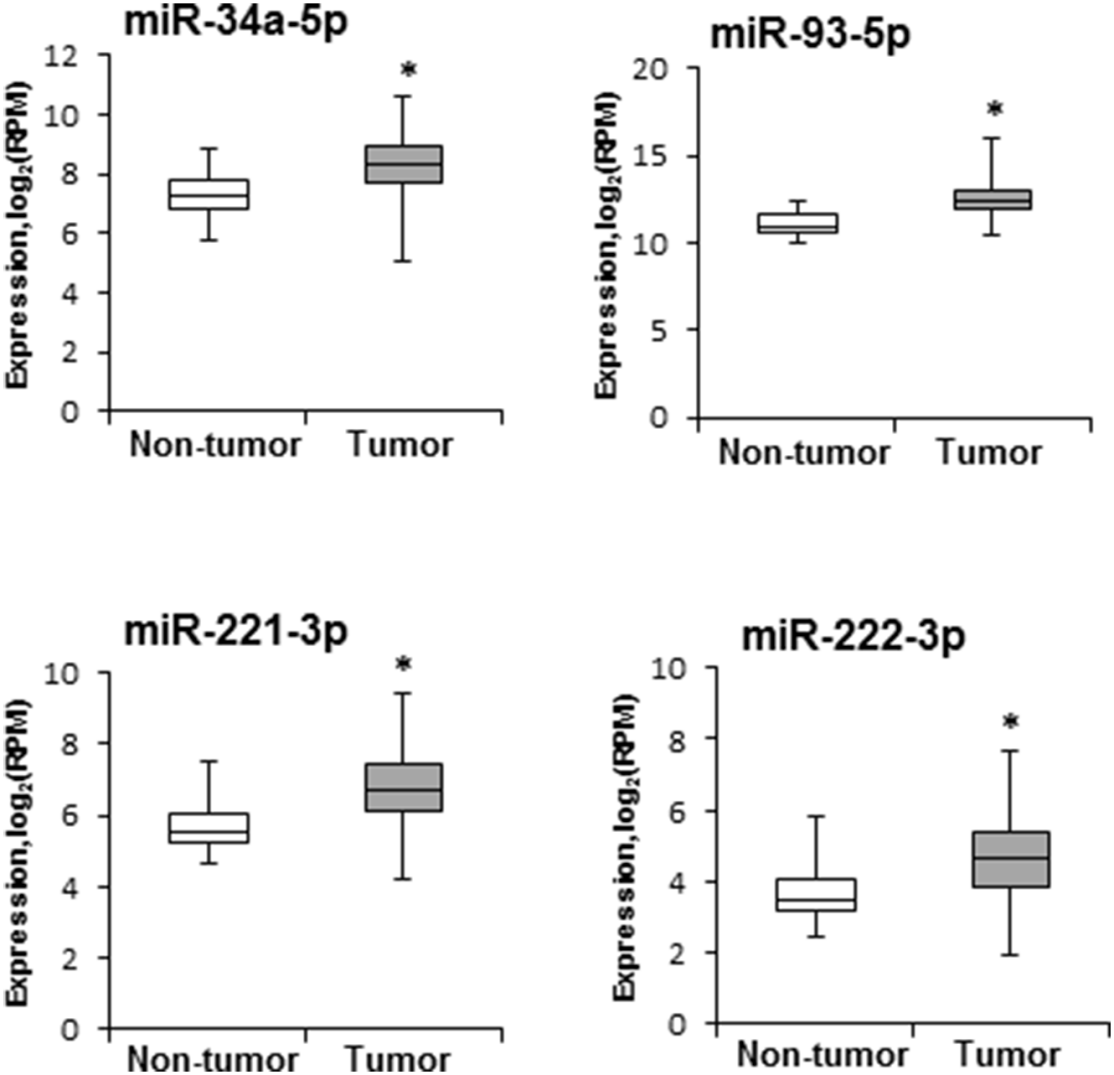
Levels of miR-34a-5p, miR-93-5p, miR-221-3p, and miR-222-3p in human HCC samples miRNA expression and clinical data were downloaded from the TCGA database (http://cancergenome.nih.gov). miRNA sequencing data are presented as a base 2-logarithm of reads per million of each miRNAs in HCC tissue samples (n = 358) relative to that in non-tumor liver samples (n = 50). The statistical analyses of miR-34a-5p, miR-93-5p, miR-221-3p, and miR-222-3p expression datasets in human HCC samples were conducted by the Mann-Whitney Rank Sum test. * - denotes a significant (P < 0.05) difference from the non-tumor liver samples.

The expression of miRNAs found to be altered in mouse NASH-derived HCC was investigated in human HCC tissue samples using data from the TCGA database. Figure 3 shows that the expression of four miRNAs, miR-34a-5p, miR-93-5p miR-221-3p, and miR-222-3p, that were over-expressed in mouse NASH-derived HCC was also significantly greater in human HCC (n = 358) as compared to non-tumor liver tissue samples (n = 50).

### Expression of the minichromosome maintenance protein 7 (MCM7) gene and its miR-106b∼25 intragenic cluster in the livers of STAM mice

**Figure 4:**
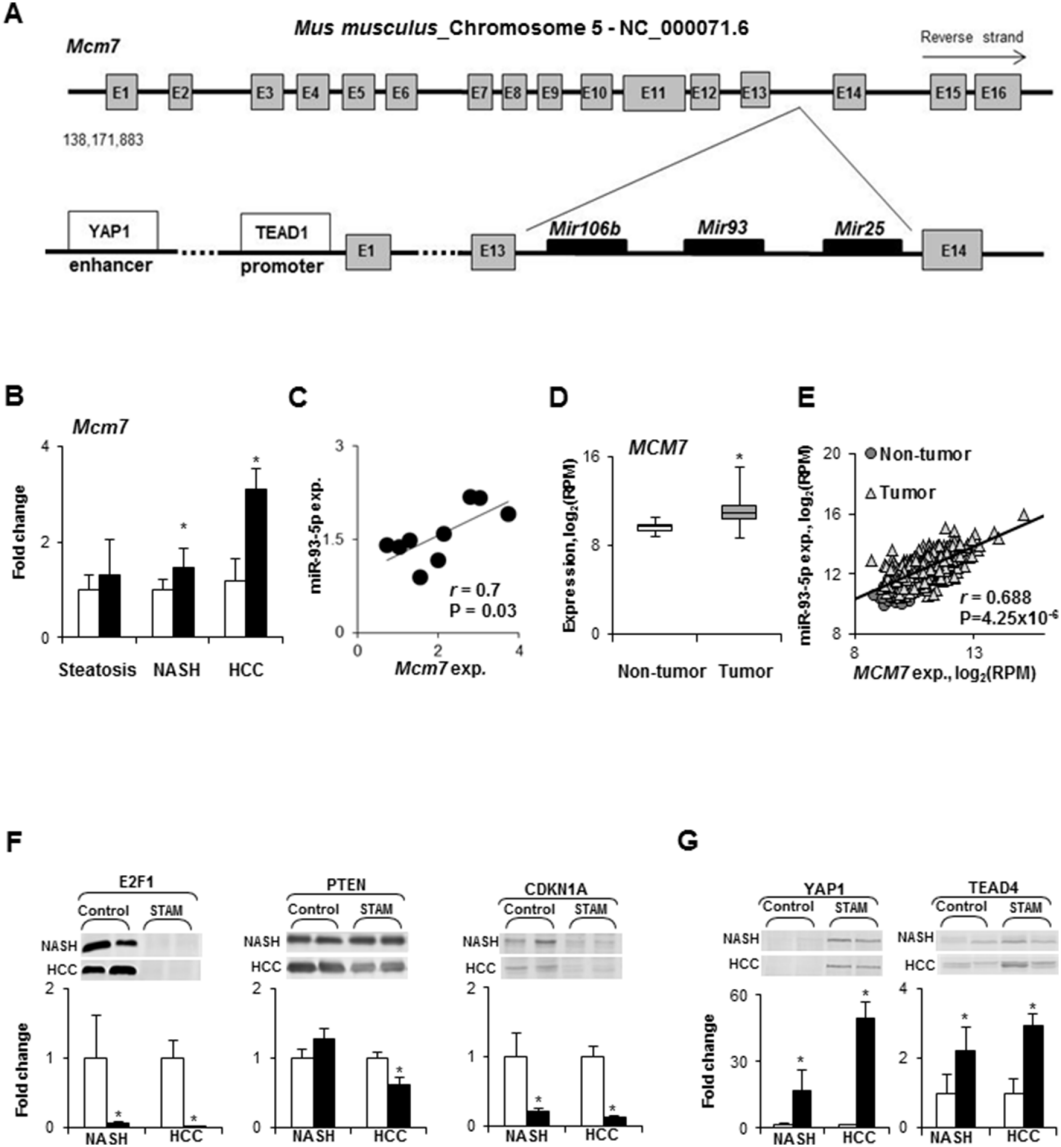
Expression of the *Mcm7* gene and miR-106b∼25 cluster during NASH-associated hepatocarcinogenesis **(A)** Diagram of the mouse *Mcm7* gene and its intragenic miR-106b∼25 cluster. **(B)** *Mcm7* expression in the livers during NASH-associate liver carcinogenesis. *White bars* – control mice; *black bars* - STAM mice. Values are mean ± SD, n = 5. * - denotes a significant (P < 0.05) difference from the age-matched control group. **(C)** Correlation plot of *Mcm7* and miR-93-5p expression during NASH-associate liver carcinogenesis. **(D)** The median expression of the *MCM7* gene in human HCC as determined by the Mann-Whitney Rank Sum test. * - denotes a significant (P < 0.05) difference from the non-tumor liver samples. **(E)** Correlation plot of *MCM7* and miR-93-5p expression in human HCC. **(F)** Level of E2F1, PTEN, and CDKN1A directly targeted by miR-106b, miR-93-5p, and miR-25 in the livers of control mice and STAM mice at NASH-fibrosis and full-fledged HCC stages of hepatocarcinogenesis. *White bars* – control mice; *black bars* - STAM mice. Values are mean ± SD, n = 5. * - denotes a significant (P < 0.05) difference from the age-matched control group. Representative Western blot images of 2 different samples from the each group are shown. **(G)** Protein level of transcriptional effectors, YAP1 and TEAD4, of the *Mcm7* gene in the livers of control mice and STAM mice at NASH-fibrosis and full-fledged HCC stages of hepatocarcinogenesis. *White bars* – control mice; *black bars* - STAM mice. Values are mean ± SD, n = 5. * - denotes a significant (P < 0.05) difference from the age-matched control group. Representative Western blot images of 2 different samples from the each group are shown.

Among the differentially expressed miRNAs in NASH-derived HCC, three miRNAs, miR-106b, miR-93, and miR-25, are members of the oncogenic miR-106b∼25 intragenic cluster [15, 16]. This cluster is highly conserved in vertebrates and is located in the 13^th^ intron of the *Mcm7* gene on mouse chromosome 5 (Figure 4A) and human chromosome 7. The miR-106b∼25 cluster is actively co-transcribed with the *MCM7* primary RNA transcript [17, 18]; hence, the expression of *Mcm7* was examined at different stages of NASH-associated liver carcinogenesis. The expression of the *Mcm7* gene was slightly, but significantly, increased in NASH/fibrotic tissue samples and markedly elevated in HCC (Figure 4B). Furthermore, Figure 4C shows that *Mcm7* expression strongly positively correlated (r = 0.70, P = 0.03) with the miR-93-5p level. Importantly, the expression of the *MCM7* gene was also increased in human HCC (Figure 4D) and positively correlated (r = 0.69, P = 4.25 x 10^-6^) with the level of miR-93-5p (Figure 4E).

To investigate the functional consequences of the miR-106b∼25 cluster over-expression with respect to the hepatocarcinogenic process, the levels of E2F1, PTEN, and CDKN1A proteins, experimentally confirmed targets of miR-106b, miR-93-5p, and miR-25, were evaluated. Figure 4F shows that the levels of E2F1, PTEN, and CDKN1A were decreased in HCC tissue samples, and E2F1 and CDKN1A in NASH/fibrotic tissue. Mechanistically, the over-expression of the miR-106b∼25 cluster during NASH-associated liver carcinogenesis may be attributed to a markedly increased level of yes-associated protein 1 (YAP1) and TEA domain transcription factor 4 (TEAD4) proteins (Figure 4G), members of YAP and TAZ transcriptional effectors of the *Mcm7* gene [16].

### Transient transfection of Hep3B HCC cells with anti-miR-93 increases cell death and drug sensitivity and decreases colony formation

**Figure 5:**
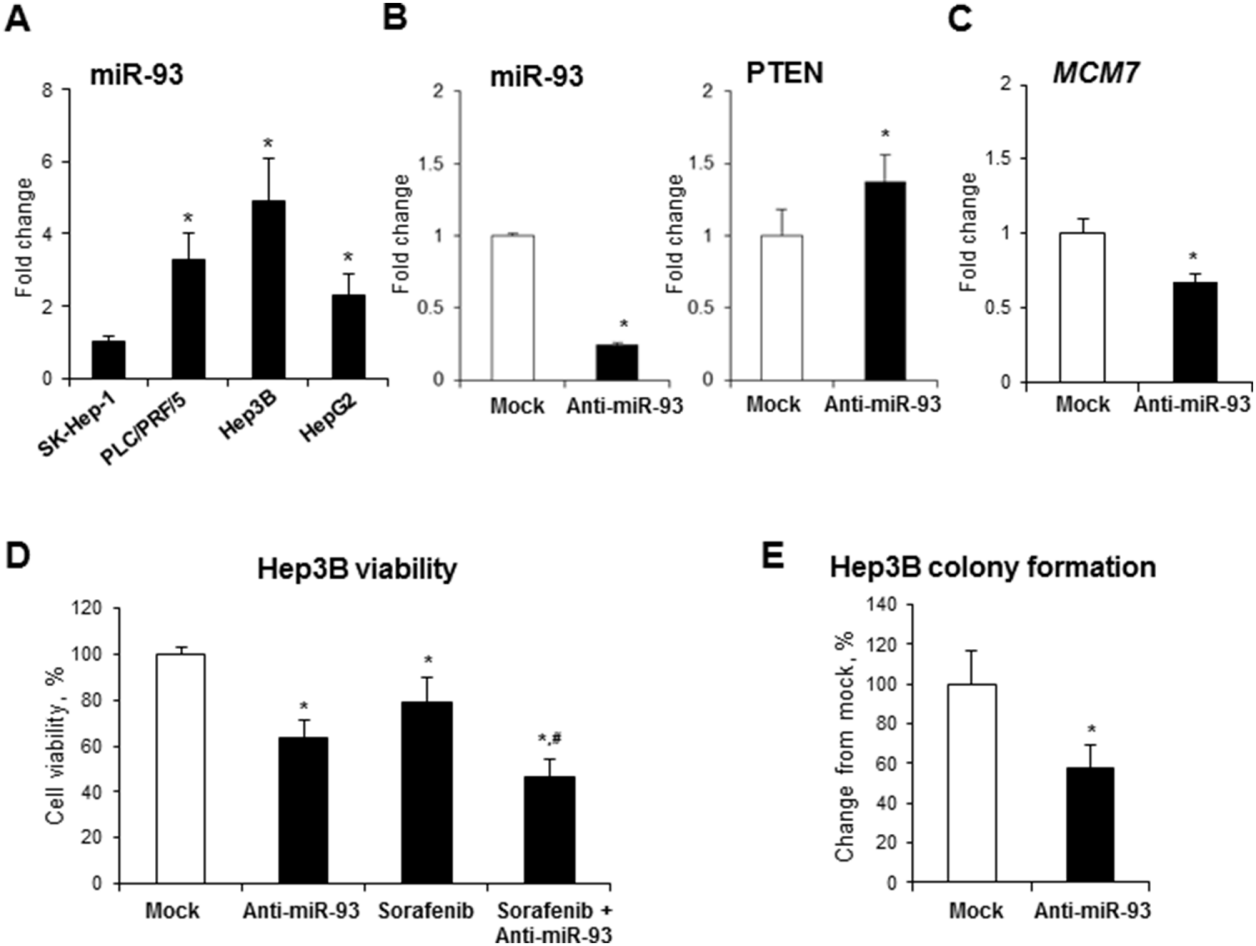
Effect of anti-miR-93 transfection on human Hep3B hepatocellular carcinoma cells **(A)** Expression of miR-93 in human liver cancer cell lines. The expression of miR-93 in α-fetoprotein-positive PLC/PRF/5, Hep3B, and HepG2 cells is presented as an average fold relative to that in α-fetoprotein-negative SK-Hep-1 cells, which were assigned a value 1. * - denotes a significant (P < 0.05) difference from SK-Hep-1 cells. **(B)** Transfection of Hep3B cells with anti-miR-93 decreased the level of miR-93 and increased the level of the experimentally confirmed miR-93 target, PTEN. **(C)** Expression of the *MCM7* gene in Hep3B cells transfected with anti-miR-93 and scrambled RNA oligonucleotide. **(D)** Effect of anti-miR-93 transfection on the viability of Hep3B and the sensitivity to Sorafenib. **(E)** Effect of anti-miR-93 transfection on the soft agar colony formation. * - denotes a significant (P < 0.05) difference from Hep3B cells transfected with scrambled RNA oligonucleotide. ^#^ - denotes a significant (P < 0.05) difference from Hep3B cells transfected with anti-miR-93 and treated with Sorafenib alone (n = 3). These results were reproduced in two independent experiments.

To determine whether or not inhibition of miR-93-5p decreases the oncogenic phenotype of cancer cells, four human liver cancer cell lines (SK-Hep-1, PLC/PRF/5, Hep3B, and HepG2) were screened for miR-93 levels (Figure 5A). Since Hep3B cells exhibited the highest level of miR-93 among the human liver cancer cell lines, this cell line was transfected with 50 nM of anti-miR-93-5p. Figure 5B shows that after the 72-hour transfection, the level of miR-93 in Hep3B cells was greatly reduced, whereas the level of tumor suppressor protein PTEN, a direct target of miR-93 [19], significantly increased. Importantly, transfection of Hep3B cells with anti-miR-93-5p reduced the expression of the *MCM7* gene (Figure 5C), cell viability (Figure 5D), increased the sensitivity to Sorafenib, a multikinase inhibitor used to treat HCC (Figure 5D), and inhibited colony formation (Figure 5E).

## DISCUSSION

In our previous study of NASH-associated liver carcinogenesis, we demonstrated extensive transcriptomic and epigenetic dysregulation of protein-coding genes and prominent alterations in cancer-related pathways during the development of NASH-derived HCC [20]. This corresponded to the well-established fact that carcinogenesis, in general, and liver carcinogenesis, in particular, are associated with extensive genomic aberrations. In addition to the primary role of numerous cancer-linked genomic alterations in the pathogenesis of HCC [11, 12], extensive research in recent years has established a significant role of miRNA deregulation in HCC [5, 21]. This has led to the suggestion that the dysregulation of miRNAome may contribute to the initiation and/or progression of hepatocarcinogenesis [22].

In the present study, we investigated alterations in the expression of hepatic miRNAs during liver carcinogenesis associated with NASH, a recently emerged HCC risk factor. We demonstrated that the development of NASH-derived HCC in mice is characterized by noticeable changes in the expression of hepatic miRNAs. In particular, we identified 10 over-expressed miRNAs (miR-17-5p, miR-221-3p, miR-93-5p, miR-25-3p, miR-181b-5p, miR-106b-5p, miR-186-5p, miR-222-3p, miR-15b-5p, and miR-223-3p; Figure 2A) that are involved in the activation of major liver carcinogenesis-related gene expression networks, especially the TGF-β- and Wnt/β-catenin signaling pathways, the roles of which are well-established in hepatocarcinogenesis [14]. These findings are in good agreement with several reports [21, 23–26] that have demonstrated altered expression of the majority of these miRNAs in human HCC. In particular, Pineau *et al.* [24] identified a signature set of 12 miRNAs that defines the development of cirrhosis-associated HCC in humans. Importantly, five of these miRNAs (miR-34a-5p, miR-93-5p, miR-106b-5p, miR-221-3p, and miR-222-3p) were in common with those in the 10-miRNA set in our study.

Among the miRNAs distinctively over-expressed in NASH-derived HCC, miR-221-3p and miR-222-3p, which exhibited a carcinogenesis stage-dependent increase in expression, and miR-25-3p, miR-93-5p, and miR-106b-5p, which are members of the oncogenic miR-106b∼25 cluster, are of special interest. Several reports have demonstrated over-expression of miR-221-3p and miR-222-3p in human HCC, which is associated with inhibition of apoptosis, activation of the TGF-β, Wnt/β-catenin, and mTOR signaling pathways, cell migration, invasion, and the formation of a more aggressive tumor phenotype [27–31]. The results of our previous [20] and present studies demonstrate deregulation of all of these molecular pathways in NASH-associated liver carcinogenesis. In contrast, it has been shown that simultaneous targeted inhibition of miR-221-3p and miR-222-3p affects multiple pro-oncogenic pathways, reverses the aggressive HCC phenotype, and suppresses tumor growth [24, 29, 32].

The miR-106b∼25 cluster is one of two paralogs of the miR-17∼92 cluster [33]. Both of these miRNA clusters are abundantly and simultaneously expressed in different cell types and are among the most potent oncogenic miRNAs that are greatly over-expressed in different types of human cancer, including HCC [15, 26, 34, 35]. The substantial overlap in function of these two miRNA clusters may be attributed, in part, to the fact that miRNA-members of these clusters, despite being located in different clusters, belong to the same miRNA families [33]. For example, miR-17-5p, located in the miR-17∼92 cluster, and miR-93-5p and miR-106b-5p, located in the miR-106b∼25 cluster, belong to the same miR-17 family [33]. Interestingly, all these three miRNAs were simultaneously over-expressed in NASH-derived HCC.

Mechanistically, the over-expression of miR-25-3p, miR-93-5p, and miR-106b-5p in NASH-derived HCC may be attributed to an increased expression of the *Mcm7* gene, which harbors the miR-106b∼25 cluster [16–18]. This suggestion is based on the results of the present study showing a concomitant up-regulation of the *Mcm7* gene and the miR-106b∼25 cluster in NASH-derived HCC, and a positive correlation between the level of miR-93-5p and the *Mcm7* transcript during mouse liver carcinogenesis and in human HCC. MCM7 is a member of the family of minichromosomal maintenance proteins that are present in all cells of eukaryotic organisms and plays an essential role in the initiation of DNA replication by ensuring that DNA replicates only once during each cell cycle [36, 37]. The results of our study show that the over-expression of *Mcm7* during NASH-associated liver carcinogenesis may be explained, at least in part, by activation of c-MYC, YAP1, Wnt/β-catenin, and ERK/MAPK signaling pathways, which was evidenced by increased protein levels of their corresponding transcription factors. This suggestion is supported by a wealth of data of a direct and co-dependent role of these transcription factors in mediating *MCM7* gene expression [16, 38–41].

The increased level of the MCM7 protein, due to gene over-expression or amplification, has been reported in various tumors [16, 17], including HCC [38]. In general, the tumorigenic effect of *MCM7* has been associated with its direct role in compromising genome integrity and, to a much greater extent, with oncogenic activity of the gene-embedded miR-106b∼25 cluster. This suggestion corresponds to the results of our study showing that over-expression of the miR-106b∼25 cluster in NASH-derived HCC, especially miR-93-5p, is accompanied by substantial decrease in the levels of E2F1, PTEN, and CDKN1A proteins, direct targets of miR-93 [19, 42, 43]. Additionally, it has been demonstrated that miR-221-3p and miR-222-3p also target PTEN and CDKN1A [44, 45], indicating that the oncogenic activity of miR-93-5p, miR-221-3p, and miR-222-3p may be attributed to the silencing of these key cancer-related genes and consequent impairment in cell-cycle arrest and inhibition of apoptosis. Indeed, in the present study, we demonstrated a substantial decrease in the levels of E2F1, PTEN, and CDKN1A proteins during the development of NASH-derived HCC, which is in a good agreement with our previous report of prominent inhibition of apoptosis during NASH-associated liver carcinogenesis [20]. Furthermore, we showed that targeted inhibition of miR-93-5p in human Hep3B HCC cells with anti-miR-93-5p reduced the oncogenic cancer cell phenotype, as evidenced by decreased cell viability, decreased colony formation, and increased sensitivity of the cells to the multikinase inhibitor Sorafenib, the only current effective systemic chemotherapeutic agent for the treatment of advanced HCC [14, 46].

In summary, the results of the present study demonstrate that NASH-associated liver carcinogenesis is characterized by substantial alterations in the expression of hepatic miRNAs, with the greatest magnitude being found in full-fledged HCC. With respect to the mechanism of liver carcinogenesis, the altered expression of miRNAs was associated with two major mechanisms that complement each other (*i*) an activation of major hepatocarcinogenesis-related pathways and (*ii*) reduced levels of miRNA targets, key components of these pathways. Importantly, several of the identified miRNAs, including miR-34a, miR-93-5p, miR-221-3p, and miR-222-3p, were also significantly over-expressed in human HCC suggesting that aberrant expression of these miRNAs may serve as an indicator of the hepatocarcinogenic process.

## MATERIALS AND METHODS

### Mouse model of *NASH*-derived carcinogenesis and liver samples

The Stelic Animal Model (STAM) of NASH-derived HCC is the first mouse model to depict the sequential evolution of clinical and pathomorphological features of the development of HCC in diabetes-associated NASH patients [9, 10]. Briefly, 2-day-old male C57BL/6J mice were injected intraperitoneally with streptozotocin (200 μg/mouse) in sterile saline buffer. Starting from 4 weeks of age, mice were continuously fed a high-fat diet (HFD-32; Clea, Tokyo, Japan). Control mice were maintained on standard animal chow. Liver samples from male STAM mice at steatotic (6 weeks), NASH-fibrotic (12 weeks), and full-fledged HCC (20 weeks) stages of liver carcinogenesis and liver samples from age-matched C57BL/6J mice were purchased from the Stelic Institute & Co., Inc. (Tokyo, Japan).

### Cell lines and cell culture

PLC/PRF/5, SK-Hep-1, HepG2, and Hep3B human liver cancer cell lines were obtained from the American Type Culture Collection (ATCC, Manassas, VA) and maintained according to the ATCC’s recommendations.

### RNA isolation and miRNAs expression profiling

Total RNA, including miRNA, was extracted from liver tissues of STAM and control mice (n = 5/group/time interval) using miRNeasy Mini kits (Qiagen, Valencia, CA) according to the manufacturer’s instructions. cDNA, generated with a miScript II RT Kit (Qiagen), was used as a template for miRNA expression analysis (n = 3/group/time interval) using Liver miFinder PCR Arrays (Qiagen). The relative amount of each miRNA was determined using the 2^*−ΔΔCt*^ method [47]. To identify miRNAs that were differentially expressed between the control and experimental groups, Benjamini-Hochberg adjusted P-values [48] were calculated to control for the false discovery rate (FDR) in multiple testing data, with an adjusted cut-off P-value of 0.1, and a fold-change threshold of 1.5.

### Bioinformatic analyses

Putative miRNA targets were identified using the Predicted Targets component of miRecords [49], an integrated resource for the analysis of animal miRNA-target interaction. This resource predicts miRNA targets produced by 11 established miRNA target prediction programs, including Diana-microT, MicroInspector, miRanda, miRTarget2, miRTarget, NbmiRTarget, PICTAR, PITA, RNA22, and TargetScan/TargetScanS. Targets predicted by a minimum of four algorithms were subjected to Ingenuity Pathways Analysis (IPA, Ingenuity Systems, Inc., Redwood City, CA). Enrichment of genes in pathways was assessed in comparison with a reference set in the Ingenuity Knowledge Base. Fisher’s exact test was used to determine the significance of pathways.

### Quantitative reverse-transcription PCR

The expression of the *MCM7* and *MIR93* genes was determined by quantitative reverse-transcription PCR (qRT-PCR) using TaqMan gene expression assays (Life Technologies, Grand Island, NY). β-Actin (*ACTB*) and RNU48 were used as endogenous controls. The relative gene expression was determined using the 2^*−ΔΔCt*^ method [47].

### Western blot analysis

Whole liver tissue lysates, containing equal quantities of proteins, were separated by 7-15% SDS-PAGE and transferred to PVDF membranes. The levels of TGF-β, CTNNB1, MAPK1, c-MYC, mTOR, AREG, E2F1, PTEN, CDKN1A, YAP1, and TEAD4 proteins were determined by Western blot analysis. IRDye 800CW-labeled anti-rabbit or IRDye 680RD-labeled anti-mouse secondary antibodies (LI-COR Biosciences, Lincoln, NE) were used for visualization. Fluorescence was measured using the Odyssey CLx Infrared Imager (LI-COR Biosciences). The images were quantified using ImageStudio 4.0 Software (LI-COR Biosciences). To control for equal loading, the relative amount of the protein of interest was normalized by staining of the membranes with REVERT Total Protein Stain (LI-COR Biosciences).

### Analysis of human miRNA and MCM7 gene expression data from online database

The *MCM7* gene and miRNA expression and clinical human HCC data were downloaded from The Cancer Genome Atlas (TCGA; http://cancergenome.nih.gov) database. All samples were median-centered and Benjamini-Hochberg adjusted P-values [48] were calculated to control the FDR in the data from human HCC samples.

### Transfection of Hep3B cells with anti-miR-93-5p

The Hep3B cells were seeded in 100 mm dishes at a density of 1 × 10^6^ cells/dish, and transfected with 50 nM of either anti-miR-93-5p (n = 3; Life Technologies) or scrambled RNA oligonucleotide (n = 3) using Lipofectamin™ 3000 transfection reagent (Life Technologies) according to the manufacturer’s instructions. Seventy-two hours post-transfection, adherent cells were harvested by mild trypsinization, washed in phosphate-buffered saline (PBS), and the viability of cells was determined with a Trypan blue exclusion assay. The cells were then re-seeded and the transfection was repeated. The viability of cells was determined at 48 hours after the second transfection. Adherent cells were then harvested by mild trypsinization, washed in PBS, and immediately frozen at -80°C for subsequent analyses. The experiments were repeated twice in triplicate.

### Cell survival analysis

To determine drug sensitivity, 72 hours after being transfected with scrambled RNA oligonucleotide or anti-miR-93, adherent Hep3B cells were harvested by mild trypsinization, washed in PBS, plated at a density of 7.5 x 10^3^ cells/well in 96-well plates, and the transfection was repeated. Cells were cultured for 24 hours and then treated with 1.0 μM Sorafenib (Santa Cruz Biotechnology, Dallas, TX), a multikinase inhibitor used for the treatment of HCC. After 48 hours of incubation, cell survival was determined using CellTiter-Glo^®^ 3D Cell Viability assay (Promega Corporation, Madison, WI).

### Soft agar colony formation assay

To investigate anchorage-independent growth potential, 72 hours after being transfected with scrambled RNA oligonucleotide or anti-miR-93, adherent Hep3B cells were harvested by mild trypsinization, washed in PBS, seeded in 100 mm dishes at a density of 1 × 10^6^ cells/dish, and the transfection was repeated. After 48 hours of incubation, 7.5 × 10^3^ viable Hep3B cells that had been transfected with either anti-miR-93 or scrambled RNA oligonucleotide were seeded onto 0.6% noble agar in growth media. After 21 days of growth, colonies were stained with 0.005% crystal violet for 1 hour, and counted using an inverted microscope Leica DMi8 (Leica Microsystems Inc., Buffalo Grove, IL) equipped with the Leica Application Suite LAS software.

### Statistical analyses

Results are presented as mean ± S.D. Student’s *t*-test was used to evaluate significant differences between STAM mice and age-matched control mice at the same time point. One-way analysis of variance (ANOVA), followed by Student-Newman-Keuls method was used to evaluate significant differences between the stages during the progression to HCC. Pearson product-moment correlation coefficients were used to determine the relationship between miR-93-5p levels and the *Mcm7* gene expression. When necessary, the data were natural log transformed before conducting the analyses to maintain a more equal variance or normal data distribution. Values of P < 0.05 were considered significant.

## SUPPLEMENTARY MATERIALS TABLE



## Abbreviations

HCC: hepatocellular carcinoma
NASH: nonalcoholic steatohepatitis
NAFLD: non-alcoholic fatty liver disease
STAM: Stelic Animal Model
miRNA: microRNA
MCM7: minichromosome maintenance protein 7
HFD: high-fat diet
