# Supplementary material for: MicroRNA deregulation in nonalcoholic steatohepatitis-associated liver carcinogenesis
Source: Oncotarget. 2017 Aug 1;8(51):88517–28. doi: 10.18632/oncotarget.19774 (PMC5687623; doi:10.18632/oncotarget.19774)
Supplement: Supplementary file 1 [file oncotarget-08-88517-s001.pdf]

MicroRNA deregulation in nonalcoholic steatohepatitis-associated liver carcinogenesis

SUPPLEMENTARY MATERIALS

Supplementary Table 1: Expression of miRNAs in the livers of STAM mice

#	miRNA	Steatosis	NASH	HCC
1	mmu-miR-34a-5p	10.4	13.2	8.1
2	mmu-miR-107-3p	2.7	4.8	3.3
3	mmu-miR-15b-5p	2.2	1.6	3.5
4	mmu-miR-322-5p	2.2	3.4	4.1
5	mmu-let-7i-5p	1.9	1.8	2.3
6	mmu-miR-106b-5p	1.8	1.7	2.3
7	mmu-miR-151-5p	1.7	1.5	1.8
8	mmu-miR-130a-3p	1.6	1.6	2.1
9	mmu-miR-152-3p	1.5	2.2	1.8
10	mmu-miR-186-5p	1.5	1.7	2.5
11	mmu-miR-27a-3p	1.3	1.8	2.1
12	mmu-miR-125a-5p	1.2	1.8	1.9
13	mmu-miR-29a-5p	1.3	1.7	1.8
14	mmu-miR-222-3p	1	1.5	2.6
15	mmu-miR-191-5p	1.3	1.6	2.2
16	mmu-miR-378a-3p	2	1.5	1.5
17	mmu-miR-199b-3p	1.5	1.6	1.7
18	mmu-miR-497a-5p	1.7	1.8	2.1
19	mmu-miR-17-5p	1.5	1	2
20	mmu-miR-19a-3p	1.7	1	1.5
21	mmu-miR-19b-3p	1.8	1.2	1.4
22	mmu-miR-199a-5p	1.6	1.4	1.3
23	mmu-miR-21a-5p	1.5	1.4	1.2
24	mmu-miR-142a-5p	1.8	1.8	2.8
25	mmu-miR-142a-3p	1.2	1.8	3
26	mmu-miR-451a	1.7	1.9	3.8
27	mmu-miR-23a-3p	1.1	1.8	1.8
28	mmu-miR-126a-5p	1.3	1.6	1.4
29	mmu-let-7b-5p	1	1.7	1.3
30	mmu-miR-223-3p	0.9	1.7	3.5
31	mmu-miR-181b-5p	0.6	0.6	2.1
32	mmu-miR-93-5p	1.4	1.2	2.1
33	mmu-miR-25-3p	1.4	1.2	2.1
34	mmu-miR-103-3p	1.4	1.5	2.1
35	mmu-miR-221-3p	0.7	1.1	2
36	mmu-miR-20a-5p	1.3	1	1.9
37	mmu-miR-20b-5p	1.2	0.9	1.8
38	mmu-miR-126a-3p	1.4	1.8	1.6
39	mmu-miR-16-5p	1.2	1.1	1.6
40	mmu-miR-24-3p	1.1	1.2	1.8

Grey box indicates fold change 1.5 and BH P value < 0.1.
